# Supplementary material for: Crosslinking Effect on Thermal Conductivity of Electrospun Poly(acrylic acid) Nanofibers
Source: Polymers (Basel). 2019 May 10;11(5):858. doi: 10.3390/polym11050858 (PMC6572130; doi:10.3390/polym11050858)
Supplement: Supplementary file 1 [file polymers-11-00858-s001.pdf]

polymers
MDPI

**Supplementary Materials**

# Crosslinking effect on thermal conductivity of electrospun poly(acrylic acid) nanofiber

**Yeongcheol Park [1], Suyeong Lee [1], Sung Soo Ha [2], Bernard Alunda [3], Do Young Noh [4], Yong Joong Lee [3], Sangwon Kim [5], and Jae Hun Seol [1,*]**

[1] School of Mechanical Engineering, Gwangju Institute of Science and Technology (GIST), Buk-gu, Gwangju 61005, Korea; young13id@gist.ac.kr (Y.P.); silver4fox@gist.ac.kr (S.L.)
[2] School of Materials Science and Engineering, Gwangju Institute of Science and Technology (GIST), 123 Cheomdangwagiro, Buk-gu, Gwangju 61005, Korea; sungsoo@gist.ac.kr
[3] School of Mechanical Engineering, Kyungpook National University, 80 Daehakro, Buk-gu, Daegu 41566, Korea; benalunda10@gmail.com (B.A.); yjlee76@knu.ac.kr (Y.J.L.)
[4] Department of Physics and Photon Science, Gwangju Institute of Science and Technology (GIST), 123 Cheomdangwagiro, Buk-gu, Gwangju 61005, Korea; dynoh@gist.ac.kr
[5] Department of Polymer Science and Engineering, Inha University, 100 Inharo, Nam-gu, Incheon 22212, Korea; sangwon_kim@inha.ac.kr
* Correspondence: jhseol@gist.ac.kr

**Diameter distributions**

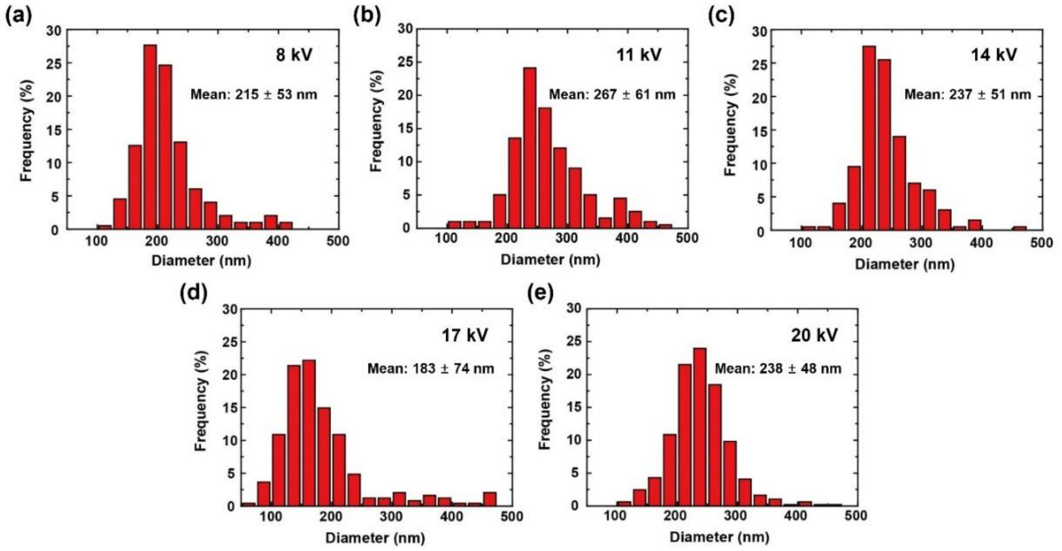


**Figure S1.** Diameter distributions of electrospun PAA NFs for various electrospinning voltages. The diameter distributions and the corresponding mean values and standard deviations of PAA NFs were manually measured from 200 PAA NFs per electrospinning voltage using ImageJ, which is an image processing program.

**Geometric change of a nanofiber *via* crosslinking**

The diameter and length changes due to crosslinking were investigated by scanning electron microscopy as shown in Fig. S2. To fix the length of the NF, 200-nm-thick platinum patterns were deposited on two contact areas, which were 500 nm wide and 830 nm long, between the NF and the two suspended membranes using a focused ion beam (FIB). The diameter slightly increased by 2.2%, and the length was almost unchanged after crosslinking. Considering the small diameter and length changes, we assumed that the diameters and the lengths were invariant after crosslinking, which may have caused an overestimation of thermal conductivity by ~4.5%. Along with the mass loss by dehydration, the volume increase of NFs results in a decrease in density, which may reduce the thermal conductivity of PAA NFs due to correspondingly increased void density [1]. Additional studies seem to be required to completely reveal the relation between the density and the degree of crosslinking in PAA NFs as well as the effect of the relation on the thermal conductivity.

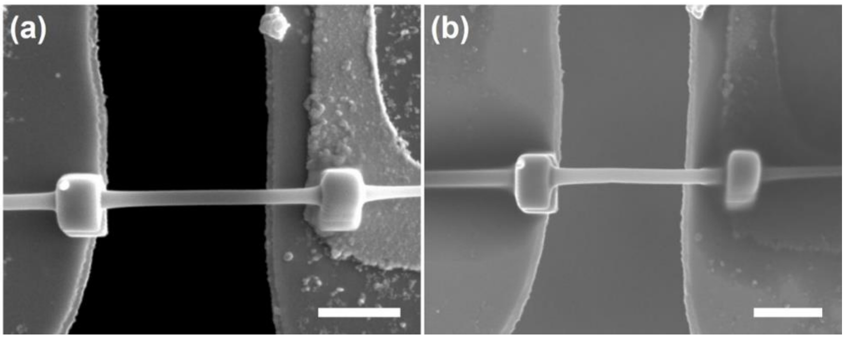


**Figure S2.** SEM images taken (a) before and (b) after crosslinking of a PAA NF. The scale bars are 1 μm.

**Table S1.** Geometric change of a PAA NF before and after crosslinking

| | As-spun | Crosslinked | Change (%) |
|---|---|---|---|
| **Diameter (nm)** | 191.2 | 195.4 | 2.2 |
| **Length (nm)** | 2694 | 2695 | 0.04 |

**Thermogravimetric analysis**

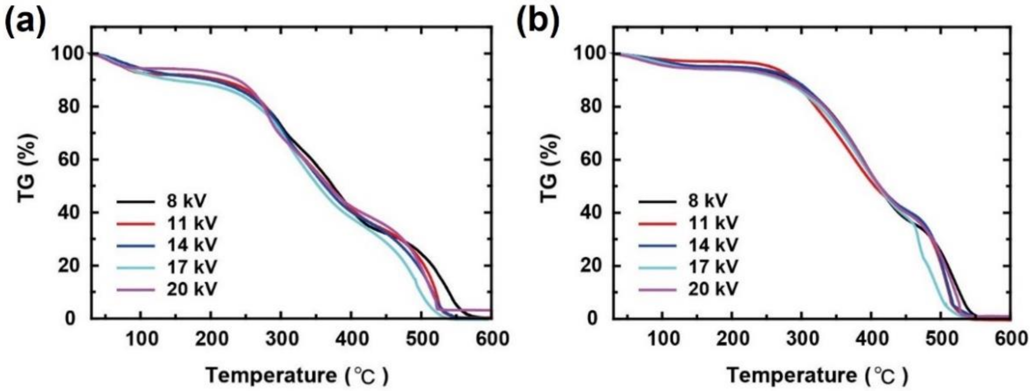


**Figure S3.** Thermogravimetric (TG) curves for (a) as-spun PAA and (b) XLPAA (crosslinked PAA) NFs for various electrospinning voltages. The first weight loss of the as-spun PAA and XLPAA NFs occurred at approximately 100 °C due to the evaporation of water molecules. After crosslinking, the second weight losses in the range of 200 to 450 °C were retarded due to the formation of intra- and intermolecular anhydride. Sequentially, the thermal decomposition of the as-spun PAA and XLPAA NFs was terminated at approximately 550 °C through the breakage of chain backbones into shorter chain fragments. The effect of electrospinning voltage on the TG curves was not observed for both as-spun PAA and XLPAA NFs.

**Polarized Raman spectroscopy analysis**

The major bands in the Raman spectrum of PAA are summarized in Table S2 [2]. As shown in Fig. S7, the spectra of both as-spun PAA and XLPAA (crosslinked PAA) NFs, which were electrospun at 14 kV, were normalized with the $CH_2$ deformation band near 1460 $cm^{-1}$ in order to compare them to each other. A prominent band was observed at 2926 $cm^{-1}$, which was assigned to $CH_2$ or CH stretching. Additionally, a C=O band arose at 1703 $cm^{-1}$. After crosslinking, the C=O band became broader and a new band appeared at 1802 $cm^{-1}$ due to the anhydride formation, which was similarly observed in the IR bands of PAA, as shown in Fig. 6 (Manuscript). Also, the modes of $CH_2$ twisting and C–$CH_2$ stretching were found at 1343 and 1108 $cm^{-1}$, respectively. While a weak band at 1185 $cm^{-1}$, which is related to C–O stretching coupled with O–H in-plane bending, was seen in the spectra of the as-spun PAA NFs, it was not observed in the spectra of the XLPAA NFs, which may be explained by the anhydride formation after crosslinking. Moreover, the spectra of the as-spun PAA NFs showed C–COOH stretching at 877 $cm^{-1}$. After crosslinking, the intensity of the C–COOH stretching band decreased, which may be ascribed to a decrease in hydrogen bonds during the crosslinking.

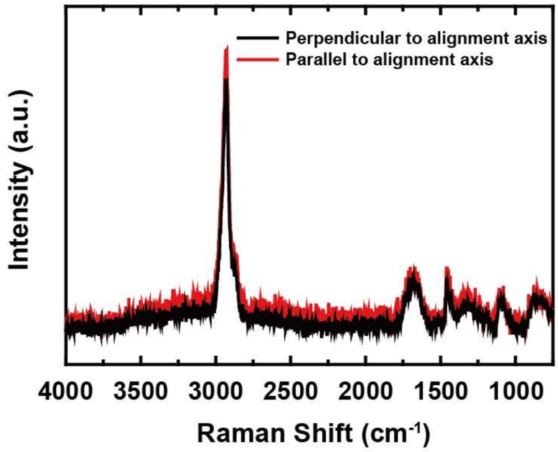


**Figure S4.** Polarized Raman spectra of an as-spun PAA mat, which was electrospun at a voltage of 14 kV. Because the numerous PAA NFs in the mat could not be perfectly aligned to the principal direction, there was no noticeable intensity difference of the band at 2935 $cm^{-1}$ due to random arrangement of PAA NFs, in contrast with the polarized Raman spectra of an as-spun PAA NF at 14 kV (Fig. 5a in the Manuscript)

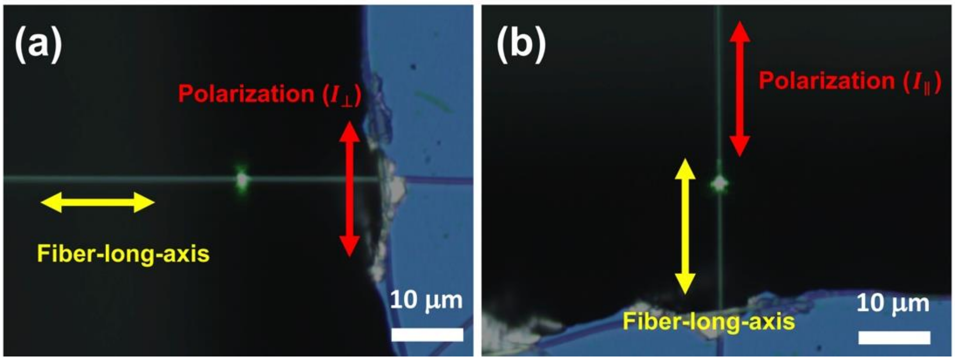


**Figure S5.** Optical microscope images, which were taken while parallelly (a) and perpendicularly (b) polarized laser beams with respect to the fiber-long-axis were focused on an individual PAA NF. The yellow and red arrows in the images indicate the directions of the fiber-long-axis and the polarization. The corresponding polarized Raman spectra were shown in Fig. 5a (Manuscript).

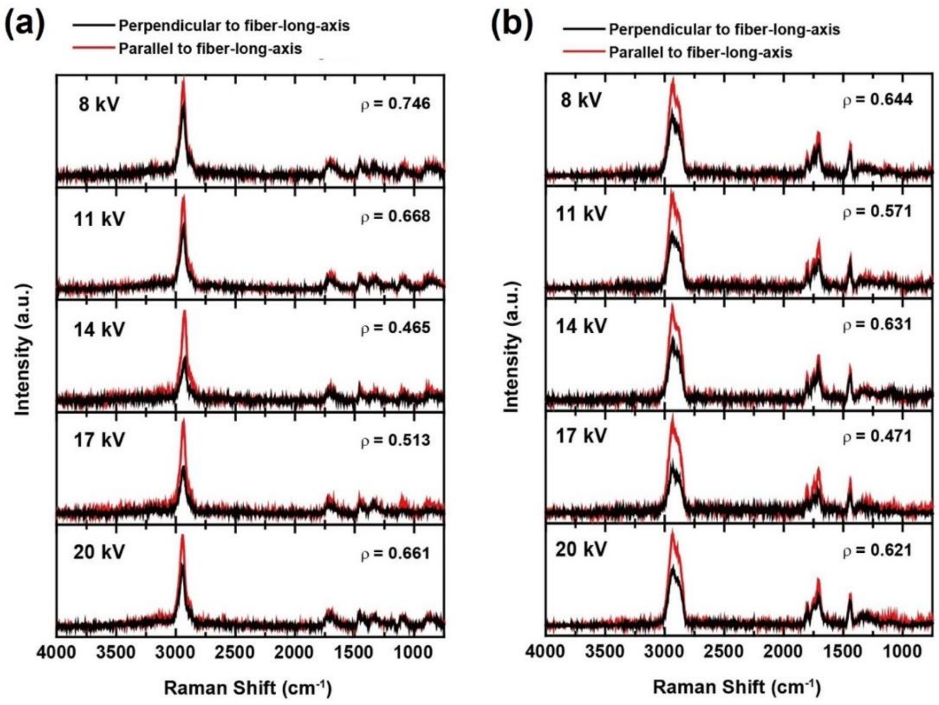


**Figure S6.** Polarized Raman spectra of (a) as-spun PAA NFs and (b) XLPAA NFs for various electrospinning voltages in a wavenumber range of 750 to 4000 cm$^{-1}$. The depolarization ratio ($\rho = I_{\perp}/I_{\parallel}$) at 2935 cm$^{-1}$ revealed that the as-spun PAA NF at 14 kV had the highest trans-gauche ratio. In contrast to the as-spun PAA NFs, the XLPAA NFs did not show any clear correlation between the depolarization ratios and the electrospinning voltages. This loss of correlation may be attributed to a decrease in the band sharpness.

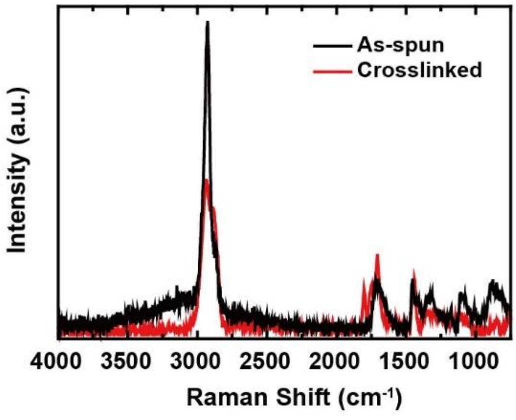


**Figure S7.** Parallel-polarized Raman spectra to incident light for an as-spun PAA NF and a XLPAA NF at 14 kV.

**Table S2.** Band assignment in Raman spectra of PAA

| Raman shift (cm$^{-1}$) | Assignments |
|---|---|
| 2926 | $CH_2$ or CH stretching |
| 1703 | C=O stretching |
| 1460 | $CH_2$ deformation |
| 1343 | $CH_2$ twisting |
| 1185 | C–O stretching coupled with O–H in-plane bending |
| 1108 | C–$CH_2$ stretching |
| 877 | C–COOH stretching |

**Fourier transform infrared analysis**

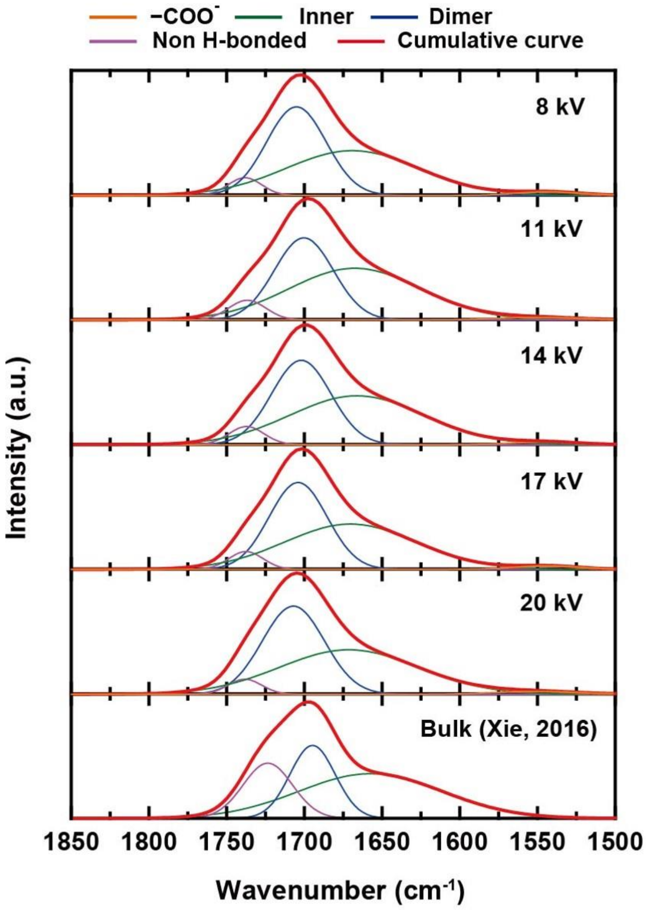


**Figure S8.** Peak deconvolution of the C=O bands in the FTIR spectra of the as-spun PAA NFs. The C=O peaks were decomposed into the several bands, which are associated with –$COO^-$ ions (1543 to 1600 $cm^{-1}$), free –COOH groups (1738 $cm^{-1}$), and hydrogen bonded –COOH groups (cyclic dimer hydrogen bond, 1705 $cm^{-1}$; inner hydrogen bond, 1669 $cm^{-1}$). There were more hydrogen bonds in the PAA NFs than in bulk PAA. Also, the electrospinning voltage did not significantly affect the quantity of hydrogen bonds. The –$COO^-$ band was hardly observed due to their negligible population.

**Table S3.** Normalized percent areas of various –COOH groups normalized with those of the C=O bands for various electrospinning voltages

| | 8 kV | 11 kV | 14 kV | 17 kV | 20 kV | Bulk (Xie, 2016) |
|---|---|---|---|---|---|---|
| **–$COO^-$** | 1.91 | 1.76 | 1.46 | 1.93 | 1.96 | - |
| **Inner** | 49.35 | 53.89 | 52.91 | 50.27 | 49.77 | 51.54 |
| **Dimer** | 43.83 | 38.53 | 40.48 | 42.62 | 44.17 | 26.51 |
| **Non H-bonded** | 4.91 | 5.82 | 5.15 | 5.18 | 4.1 | 21.95 |

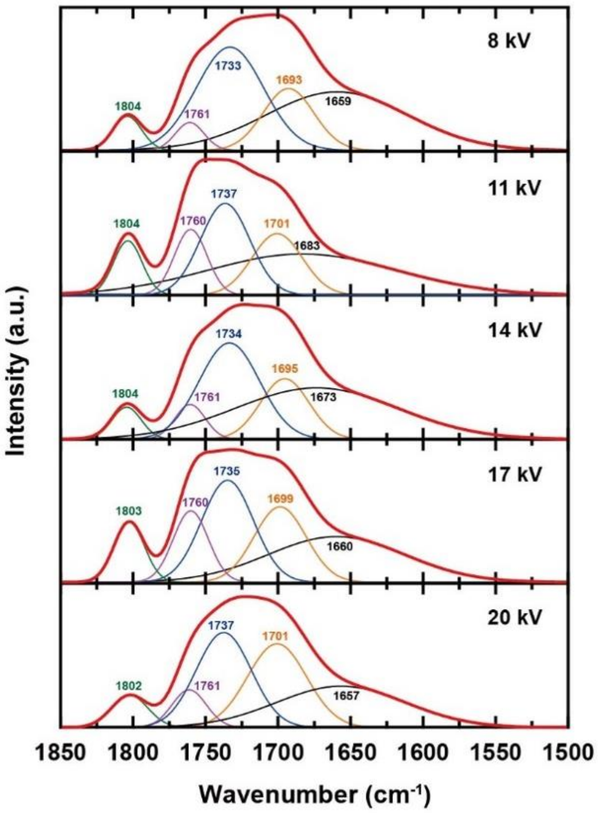


**Figure S9.** Peak deconvolution of the C=O bands ranging from 1500 to 1850 $cm^{-1}$ for investigating the anhydride structures. The bands at 1803, 1761, and 1734 $cm^{-1}$ indicate that glutaric and isobutyric anhydrides are present in the XLPAA NFs [3]. However, there is no evidence for the presence of succinic anhydride.

**X-ray diffraction analysis**

XRD analysis was conducted to study the molecular structures of the as-spun PAA and XLPAA NFs, which were electrospun at various electrospinning voltages. As shown in Fig. S10, the PAA NFs had a broad and amorphous peak at approximately $2\theta = 20^{\circ}$ with a shoulder on the right side of the peak, which is consistent with previous XRD characterizations [4–6].

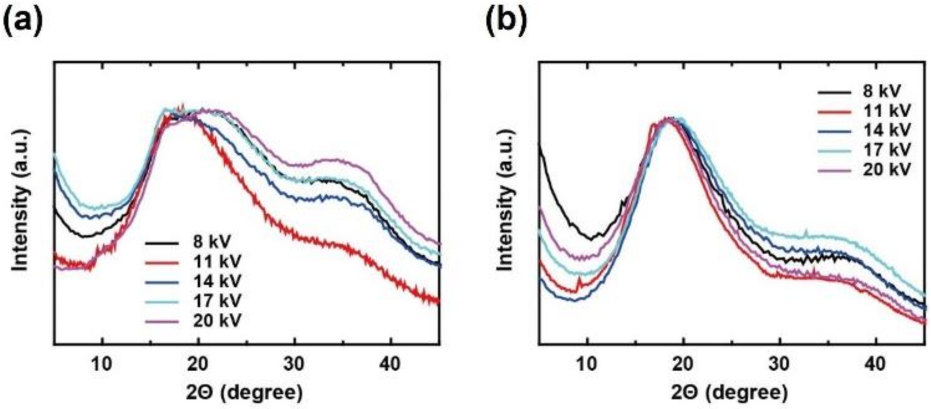


**Figure S10.** X-ray diffraction analysis for the (a) as-spun PAA and (b) XLPAA NFs for various electrospinning voltages. The as-spun PAA NFs had amorphous characteristics having a broad peak at $2\theta = 20^{\circ}$ with a shoulder.

**Measurement method**

The thermal conductivity of the electrospun PAA NFs was measured using suspended microdevices [7]. Considering the low thermal conductance of polymer nanofibers, the differential bridge method was applied to enhance the measurement sensitivity [8]. However, the background thermal conductance cannot be eliminated with the differential bridge method. The background conductance, which occurs through residual gas conduction, radiation, and the underlying substrate, is only on the order of 0.1 nW/K at room temperature. However, it cannot be neglected for a sample with a low thermal conductance. A recent study successfully removed the background conductance by applying the same heat energies to both the suspended microdevices, which were located in a cryostat, with and without a sample [9]. This idea was also applied to the differential bridge method [10], which was adopted for our measurements changing the locations of the resistances and the suspended microdevices as shown in Fig. S11.

Taking into account the location change, the sample thermal conductance was derived as follows. According to the relationship between voltages and resistances in the Wheatstone bridge circuit, the gate voltage ($v_g$) to circuit-driving source voltage ($v_{AC,s}$) is given as

$$\frac{v_g}{v_{AC,s}} = \frac{R_s}{R_s+R_1} - \frac{R_{s,ref}}{R_{s,ref}+R_2}, \tag{S1}$$

where $R_1$ is a 5-kΩ precision resistor (Vishay, Y10735K00000T9L), $R_2$ is a potentiometer, and $R_s$ and $R_{s,ref}$ are the resistances of platinum resistance thermometers (PRTs) on the sensing membranes with and without an NF, respectively. Here, $R_1$ and $R_2$ were assumed to be constants because they were located in the constant room temperature environment outside the cryostat, whereas $R_s$ and $R_{s,ref}$ varied with the temperature inside the cryostat. Considering the variation of the resistances due to Joule heating during the measurement, Eq. S1 becomes

$$\frac{v_g}{v_{AC,s}} = \frac{R_s(1+\alpha+\beta)}{R_s(1+\alpha+\beta)+R_1} - \frac{R_{s,ref}(1+\alpha)}{R_{s,ref}(1+\alpha)+R_2} = \frac{1+\alpha+\beta}{(1+\alpha+\beta)+R_1/R_s} - \frac{1+\alpha}{(1+\alpha)+R_2/R_{s,ref}}, \tag{S2}$$

where $\alpha$ and $\beta$ are the resistance increases with the temperature rises through the background and sample thermal conductances, respectively. When $v_g$ approaches zero, $\frac{R_1}{R_s} \approx \frac{R_2}{R_{s,ref}}$ is limited to a constant ($a$). Then, Eq. S2 becomes

$$\frac{v_g}{v_{AC,s}} = \frac{1+\alpha+\beta}{(1+\alpha+\beta)+a} - \frac{1+\alpha}{(1+\alpha)+a}. \tag{S3}$$

If $(1+\alpha)$, $\beta$, and $\frac{1+\alpha}{(1+\alpha)+a}$ are denoted as $x$, $\Delta x$, and $y$ $(\equiv x/(x+a))$, respectively, Eq. S3 is rewritten as

$$\frac{v_g}{v_{AC,s}} = (y+\Delta y) - y = \Delta y. \tag{S4}$$

Because small temperature variations occur through background and sample thermal conductances ($\alpha, \beta \ll 1$), $\Delta y$ is approximated to $\Delta x y'$. Therefore, excluding the background thermal conductance, we obtain a relation between $v_g$ and the sample thermal conductance in this setup:

$$v_g \approx v_{AC,s}\left\{\beta \cdot \frac{a}{(1+a)^2}\right\}. \tag{S5}$$

After the $\beta$ values were obtained from the measured $v_g$ values, the increases in resistances and the corresponding temperature changes were calculated; thus, the thermal conductance of the sample were obtained. This derivation confirms that the measurement setup of the current study is basically the same as that of the previous study [10].

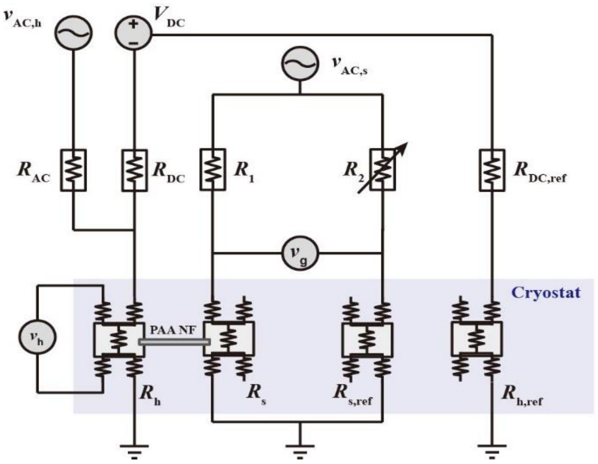


**Figure S11.** Schematic of the measurement setup. In this setup, $v_{AC,h} \approx 5$ V, $v_{AC,S} \approx 0.008$ V, $R_{AC} \approx 10$ MΩ, $R_{DC} \approx R_{DC,ref} \approx 500$ kΩ, and $R_1 \approx 5$ kΩ. Before the measurement, $R_2$ was adjusted to be in the range of 4.6 to 5.5 kΩ, which corresponded to a $v_g$ range of 1 to 10 μV.

**Measurement results**

The effect of molecular structure on the thermal conductivity of a PAA NF was investigated in this study. As explained in the Manuscript, the molecular structure of a PAA NF was changed by temperature variation. After the thermal conductivity of an as-spun PAA NF was measured, the NF was annealed at 127 °C, and then that of the $T_g$-annealed NF was measured. Subsequently, the NF was annealed at 217 °C, at which the molecular chains became crosslinked, and the thermal conductivity of the crosslinked NF was measured. Figure S12 shows the measurement results of ten NFs, which were electrospun at various voltages. In addition to these thermal conductivity results measured in the entire temperature range, the thermal conductivity of two additional PAA NFs per electrospinning voltage were measured at 27 °C and the results are presented in Fig. S13, which shows the thermal conductivity of the twenty PAA NFs as a function of diameter in total. However, we did not observe a diameter dependence of the thermal conductivity of the PAA NFs. All of the measured thermal conductivity values at 27 °C are listed in Table S4.

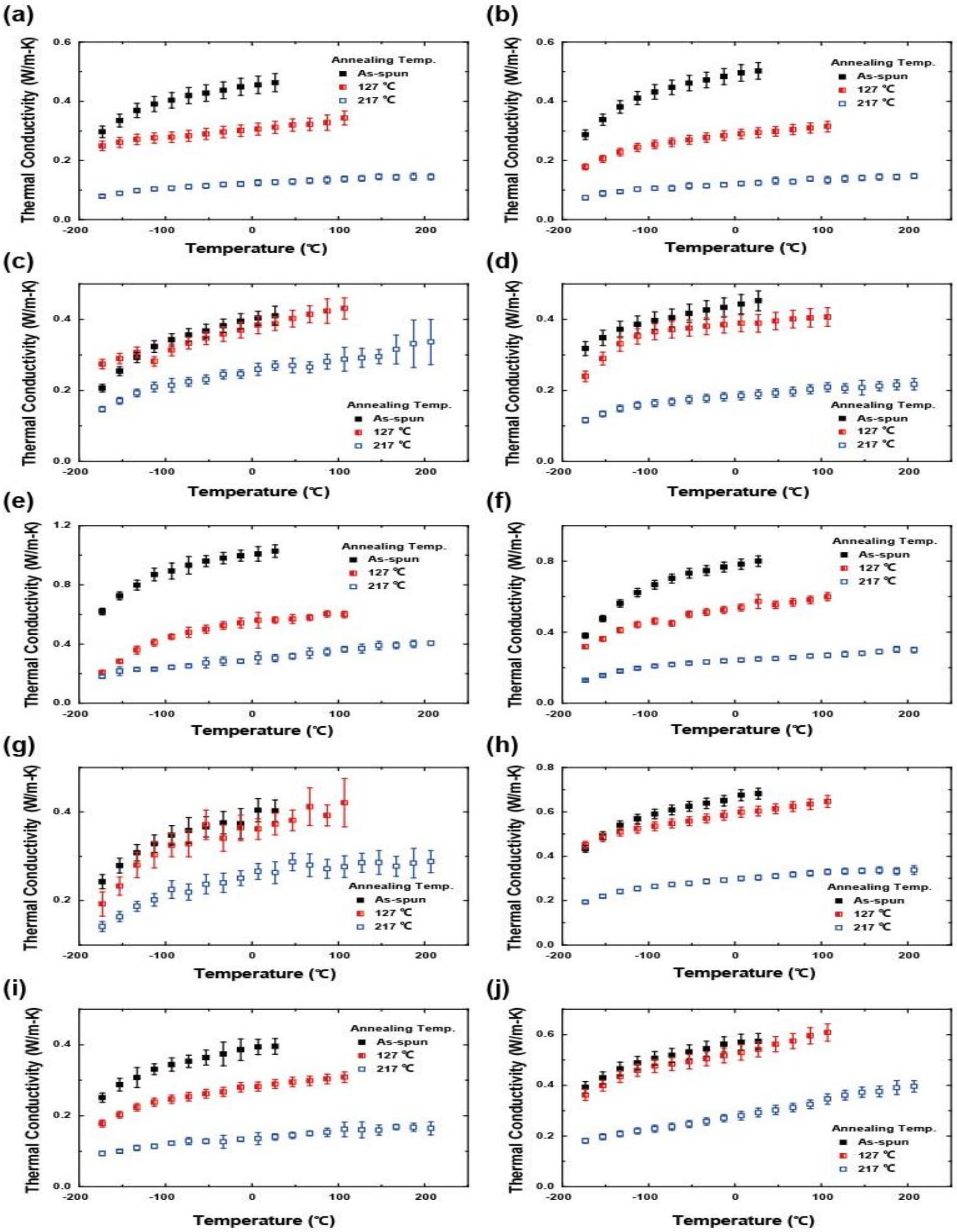


**Figure S12.** Thermal conductivities of PAA NFs, which were electrospun at various voltages, with various diameters. The diameters and the electrospinning voltages of the PAA NFs are as follows: (a) 8 kV, 191 nm, (b) 8 kV, 228 nm, (c) 11 kV, 184 nm, (d) 11 kV, 206 nm, (e) 14 kV, 152 nm, (f) 14 kV, 159 nm, (g) 17 kV, 162 nm, (h) 17 kV, 216 nm, (i) 20 kV, 156 nm, and (j) 20 kV, 159 nm.

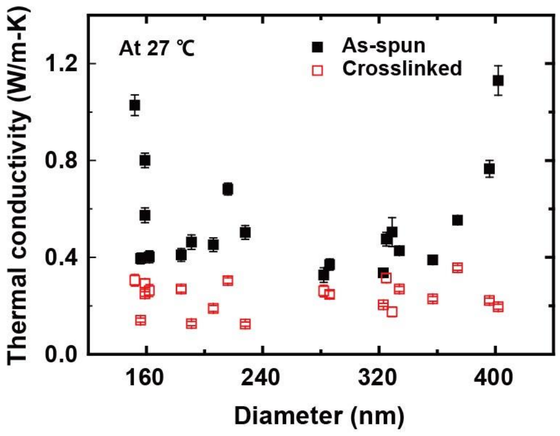


**Figure S13**. Thermal conductivity of the as-spun PAA and XLPAA NFs as a function of diameter at room temperature. We did not observe any diameter dependence of thermal conductivity from the measurement results of both the as-spun PAA and XLPAA NFs, which may be due to whipping instability in the electrospinning process [11].

**Table S4.** Thermal conductivity of the as-spun PAA and XLPAA NFs for various electrospinning voltages and diameter at room temperature.

| Electrospinning voltage (kV) | Diameter (nm) | Thermal conductivity (W/m-K) | | |
|---|---|---|---|---|
| | | As-spun | $T_g$-annealed | Crosslinked |
| 8 | 191 | 0.463 ± 0.030 | 0.312 ± 0.021 | 0.127 ± 0.009 |
| | 228 | 0.503 ± 0.028 | 0.294 ± 0.017 | 0.124 ± 0.008 |
| | 323 | 0.336 ± 0.010 | - | 0.204 ± 0.006 |
| | 329 | 0.504 ± 0.060 | - | 0.175 ± 0.020 |
| 11 | 184 | 0.410 ± 0.027 | 0.388 ± 0.020 | 0.269 ± 0.013 |
| | 206 | 0.452 ± 0.028 | 0.389 ± 0.025 | 0.189 ± 0.012 |
| | 357 | 0.389 ± 0.016 | - | 0.228 ± 0.010 |
| | 396 | 0.765 ± 0.035 | - | 0.222 ± 0.010 |
| 14 | 152 | 1.028 ± 0.043 | 0.563 ± 0.021 | 0.305 ± 0.024 |
| | 159 | 0.800 ± 0.030 | 0.573 ± 0.039 | 0.249 ± 0.010 |
| | 374 | 0.553 ± 0.017 | - | 0.357 ± 0.010 |
| | 402 | 1.130 ± 0.061 | - | 0.196 ± 0.011 |
| 17 | 162 | 0.402 ± 0.024 | 0.372 ± 0.024 | 0.263 ± 0.025 |
| | 216 | 0.682 ± 0.025 | 0.603 ± 0.022 | 0.304 ± 0.011 |
| | 282 | 0.327 ± 0.030 | - | 0.261 ± 0.024 |
| | 286 | 0.370 ± 0.023 | - | 0.247 ± 0.015 |
| 20 | 156 | 0.396 ± 0.022 | 0.289 ± 0.013 | 0.141 ± 0.009 |
| | 159 | 0.574 ± 0.030 | 0.542 ± 0.030 | 0.293 ± 0.017 |
| | 325 | 0.475 ± 0.028 | - | 0.314 ± 0.019 |
| | 334 | 0.427 ± 0.017 | - | 0.269 ± 0.011 |

## References


1. Yu, S.; Park, C.; Hong, S.M.; Koo, C.M. Thermal conduction behaviors of chemically cross-linked high-density polyethylenes. *Thermochim. Acta* **2014**, *583*, 67–71.
2. Dong, J.; Ozaki, Y.; Nakashima, K. Infrared, Raman, and near-infrared spectroscopic evidence for the coexistence of various hydrogen-bond forms in poly(acrylic acid). *Macromolecules* **1997**, *30*, 1111–1117.

3. McGaugh, M.C.; Kottle, S. The thermal degradation of poly(acrylic acid). *J. Polym. Sci. Part B Polym. Lett.* **1967**, *5*, 817–820.
4. Nam, S.Y.; Lee, Y.M. Pervaporation and properties of chitosan-poly(acrylic acid) complex membranes. *J. Memb. Sci.* **1997**, *135*, 161–171.
5. Sun, S.; Chen, X.; Liu, J.; Yan, J.; Fang, Y. A Novel Two-Component Physical Gel Based on Interaction Between Poly(acrylic acid) and 6-Deoxy-6-amino-b-cyclodextrin. *Polym. Eng. Sci.* **2009**, 99–103.
6. Shanker, A.; Li, C.; Kim, G.-H.; Gidley, D.; Pipe, K.P.; Kim, J. High thermal conductivity in electrostatically engineered amorphous polymers. *Sci. Adv.* **2017**, *3*, 1–8.
7. Shi, L.; Li, D.Y.; Yu, C.H.; Jang, W.Y.; Kim, D.; Yao, Z.; Kim, P.; Majumdar, A. Measuring thermal and thermoelectric properties of one-dimensional nanostructures using a microfabricated device. *J. Heat Transf.* **2003**, *125*, 881–888.
8. Wingert, M.C.; Chen, Z.C.Y.; Kwon, S.; Xiang, J.; Chen, R. Ultra-sensitive thermal conductance measurement of one-dimensional nanostructures enhanced by differential bridge. *Rev. Sci. Instrum.* **2012**, *83*, 024901.
9. Weathers, A.; Bi, K.; Pettes, M.T.; Shi, L. Reexamination of thermal transport measurements of a low-thermal conductance nanowire with a suspended micro-device. *Rev. Sci. Instrum.* **2013**, *84*, 084903.
10. Zeng, X.; Xiong, Y.; Fu, Q.; Sun, R.; Xu, J.; Xu, D.; Wong, C.-P. Structure-induced variation of thermal conductivity in epoxy resin fibers. *Nanoscale* **2017**, *9*, 10585–10589.
11. Ma, J.; Zhang, Q.; Mayo, A.; Ni, Z.; Yi, H.; Chen, Y.; Mu, R.; Bellan, L.M.; Li, D. Thermal conductivity of electrospun polyethylene nanofibers. *Nanoscale* **2015**, *7*, 16899–16908.
